# A systematic review and meta-analysis on the prevalence of mental disorders among children and adolescents in Europe

**DOI:** 10.1007/s00787-022-02131-2

**Published:** 2022-12-30

**Authors:** Rosemarie Sacco, Nigel Camilleri, Judith Eberhardt, Katja Umla-Runge, Dorothy Newbury-Birch

**Affiliations:** 1 School of Social Sciences, Humanities and Law, Teesside University, Middlesbrough, UK; 2 School of Medicine, Cardiff University, Cardiff, Wales; 3 School of Medicine and Surgery, University of Malta, Msida, Malta; 4 Mental Health Services, Attard, Malta

**Keywords:** Prevalence, Child and adolescent, Mental disorders, Europe

## Abstract

Most mental disorders appear by age 14, but in most cases, they remain undiagnosed and untreated well into adulthood. A scoping review showed an absence of systematic reviews that address prevalence rates of mental disorders among children and adolescents in Europe that are based on community studies conducted between 2015 and 2020. To estimate the updated pooled prevalence of Anxiety Disorder, Depressive Disorder, Attention Deficit Hyperactivity Disorder (ADHD), Conduct Disorder (CD), Oppositional Defiant Disorder (ODD), Autism Spectrum Disorder, Eating Disorders, Substance Use Disorders (SUD), among children and adolescents living in Europe, a search strategy was conducted using MEDLINE, Embase and Psych Info and studies were also identified from reference lists and gray literature. Eligible studies were evaluated for reliability, validity, and bias. Trends of prevalence rates for each mental disorder were calculated. Almost one in five young people in Europe were found to suffer from a mental disorder, with a pooled prevalence rate of 15.5%. Anxiety disorders had the highest pooled prevalence rate (7.9% (95% CI 5.1–11.8%, *I*^2^: 98.0%)), followed by ADHD (2.9% (95% CI 1.2–6.9%, *I*^2^ = 94.3%)), ODD (1.9% (95% CI 1.0–3.7%, *I*^2^ = 98.4%)), depressive disorder (1.7% (95% CI 1.0–2.9%, *I*^2^ = 97.7%)), CD (1.5% (95% CI 0.6–3.8%, *I*^2^ = 98.8%)) and ASD (1.4% (95% CI 0.4–5.4%, *I*^2^ = 99.7%). No studies on SUD were identified. The mental health of children and adolescents may be improved by introducing routine screening, refining diagnostic sensitivity, raising awareness of mental disorders, minimizing stigma and socioeconomic inequality, as well as developing early intervention services. These facilitators of good mental health need to be prioritized, especially at a time of unprecedented risk factors for poor mental health.

**Supplementary Information:**

The online version contains supplementary material available at 10.1007/s00787-022-02131-2.

## Introduction

Mental disorders (MD) are characterized by considerable disturbance in a person’s emotion regulation, cognition, or behavior, severe enough to impair function in important areas [1].The majority of MD in the general population appear by age fourteen (1, 2). Mental disorders which commonly present in childhood include neurodevelopmental disorders, such as Attention Deficit Hyperactivity Disorder (ADHD) and Autism Spectrum Disorder (ASD), depressive, anxiety, and eating and substance use disorders [2]. Most of these remain undiagnosed and untreated well into adulthood [3]. Reasons for this include stigma [3], parental [4] and teacher [5] difficulty in recognizing mental disorders, lack of screening programs and awareness campaigns [4], limited service availability [3] and long waiting lists with poor access to services [3]. Untreated childhood mental disorders are linked to short- and long-term morbidity and mortality [6]. Young people with untreated mental disorders may have difficulty functioning in all areas of life, resulting in problems such as impaired ability to reach their educational potential and difficulty maintaining friendships [7]. Moreover, when untreated mental disorders persist into adulthood, there is a higher risk of restricted occupational opportunities [7], need for social benefits [8] and involvement in criminality [7, 9], resulting in higher levels of morbidity and mortality [10] as well as negatively affecting the society at large.

Developing and improving services to enhance early identification and effective treatment of childhood MDs are a public health priority [11] since improvement in this regard would have a positive impact on all of society, both now and in future [11]. Such service development is best informed by epidemiological studies to ensure that the present needs are met [12]. Register-based and population studies measuring the prevalence of mental disorders among young people have been increasingly conducted in various parts of the world [14–14]. While these community studies present important findings, systematic reviews (SR) are needed to appraise such literature and provide cross-national comparisons of prevalence rates, thereby revealing prevalence variability across countries. This may help elucidate the unique needs and define priorities for specific regions.

A scoping literature search using PubMed and Google Scholar databases, identified a number of systematic reviews on the prevalence of young people, which had been published since 2015. Most of these focused on subpopulations such as the child welfare system [15] or specific mental disorders such as ADHD in Italy [16] and globally [17, 18], and ASD among immigrants in Europe [19] and in the global general population [20]. While these focused reviews may be useful for a more in-depth understanding of these specific disorders, they do not present a complete picture of the prevalence of mental disorders in Europe. Another study published in 2015 presented a comprehensive meta-analysis of the prevalence of a range of mental disorders among young people worldwide [14]. This identified 48 studies published as early as 1987. Although this large time span helped to increase its statistical power, its results may not reflect the present situation, when considering the rising trend in the prevalence of mental disorders over time [21, 22]. Moreover, prevalence may vary considerably across continents with varying income and developing status therefore a global prevalence rate may not necessarily provide an accurate picture of the European situation [23].

A number of gaps were identified in the literature from the above-mentioned search. First, none of the identified systematic reviews presented data on anxiety, mood and substance use disorders among young people in European countries. Furthermore, the identified systematic reviews did not include original articles published after 2015. An urgent update on the prevalence of mental disorders in Europe is therefore needed since prevalence rates have shown a rising trend over time [21, 22], possibly due to dynamic risk factors, such as improved neonatal care [24], maternal substance misuse [25], childhood stress [26, 27] as well as evolving diagnostic criteria [28, 29]. There are prominent differences between today’s gold standard diagnostic criteria (such as the Diagnostic Statistical Manual 5th Edition (DSM-V)) compared to outdated ones (such as the Diagnostic Statistical Manual 3rd Edition (DSM-3)), so individuals who would not have previously met criteria for a MD may now obtain a diagnosis and vice versa. Consequently, original articles that used outdated diagnostic criteria or that were published several years ago may not accurately demonstrate the present situation. This review sets out to address this gap in the literature by providing a comprehensive and updated picture of the prevalence of mental disorders among young people in Europe based on original studies published since 2015.

## Aims

This SR aimed to:

1. Present prevalence rates (published between 2015 and 2020) of mental disorders (Anxiety Disorder, Depressive Disorder (DD), Attention Deficit and Hyperactivity Disorder (ADHD), Conduct Disorder (CD), Oppositional Defiance Disorder (ODD), Autism Spectrum Disorder (ASD), Eating Disorders (ED) and Substance Use Disorders (SUD)) that meet DSM-IV, DSM-V or ICD-10 criteria, among 5- to 18-year-olds in Europe;
2. Compare prevalence rates among various countries in Europe;
3. Compare prevalence rates across gender and level of education; and
4. Use the results to produce recommendations for service development and research priorities.

## Methods

### Literature search and search strategy

A literature search was conducted on PubMed and Google Scholar to review the existing studies and identify that there was a gap in the literature on the prevalence of mental disorders among young people in Europe. PROSPERO [30], an international database of registered systematic reviews, was searched to determine that there were no ongoing or published reviews in this area.

A protocol for this study was developed and registered on Prospero (Registration number: CRD42020210451). A search strategy (Supplement 1) was created by one author (RS) using the SPIDER model [31] and peer reviewed by two other authors (NC, KUR). The searches to identify relevant literature published since 2015 was conducted on MEDLINE, Embase and PsychInfo databases. The gray literature was searched using EthOS, SCOPUS and an advanced Google Search. All the literature found by April 30th 2020 was exported to Mendeley.

### Eligibility criteria

The inclusion criteria were: (i) Studies providing data on the following mental disorders: ASD (including Asperger’s), ADHD (including Attention Deficit Disorder), CD, ODD, DD (including depressive episode, major depressive disorder, persistent depressive disorder, dysthymia, disruptive mood dysregulation disorder), Anxiety Disorder (including general anxiety disorder, panic disorder, social anxiety, agoraphobia, phobic disorders, obsessive compulsive disorder), eating disorders (including anorexia nervosa, bulimia nervosa, binge eating disorders), substance use disorders (including alcohol and drug-use disorders), (ii) Epidemiological studies which determine the prevalence of the disorders listed above, (iii) Participants aged 5–18 years, (iv) European or transcontinental countries partly located in Europe, (v) Original research article, vi. Published in 2015 or later, (vii) English language used for abstract, (viii) ICD-10, DSM-IV or DSM-V diagnostic criteria are used. The exclusion criteria were: (i) Studies focusing on minority groups rather than the general population, (ii) Studies which are not original research (such as systematic reviews and meta-analyses), (iii) Studies which do not use the diagnostic criteria specified in the inclusion criteria, (iv) Studies which do not include European or transcontinental countries that are partly located in Europe, (v) Studies published before 2015.

### Study identification and selection procedures

All titles and abstracts were reviewed by one author (RS) and 20% of these were checked independently by DNB. Abstracts were selected if they satisfied the following criteria: (i) Original prevalence studies on mental disorders, (ii) Included participants aged 5–18 years, (iii) Included European countries or transcontinental countries that are partly located in Europe, (iv) Published from 2015 onwards. An over-inclusive approach was used so when abstracts did not have enough information to determine eligibility, they were still selected for further review. Any disagreements between the two authors were resolved through discussions between RS and DNB. RS reviewed all full-text articles of the selected abstracts and determined their eligibility based on the inclusion and exclusion criteria listed above. Twenty percent of the full-text articles were checked independently by DNB and any disagreements were resolved through discussions between the two authors. This process was not pilot tested. Reference lists of included studies were also checked to identify other potentially eligible studies. Authors of studies with unreported data information were emailed up to two times, to ask for this.

### Data extraction and quality analysis

For each mental disorder, the following data were extracted from the selected studies and inputted into a Microsoft Excel spreadsheet: country, region/nationwide, target population, age range, level of education (primary. secondary school, both), diagnostic classification system (ICD-10, DSM-IV, DSM-V), study type (population study/ register-based study), register, number of study phases, sampling method, screening tools, response rate for screening phase, diagnostic tools, response rate for diagnostic phase, date published, sample size, total number of events (total number of individuals diagnosed with the specific disorder), total number of events among males, total number of males in sample size, total number of events among females, total number of females in sample size, total number of events among primary school children, total number of primary school children in sample size, total number of events among secondary school children, total number of secondary school children in sample size. The age and country of participants were used to determine the level of education from other online sources when this was not mentioned in the original article. The Risk of Bias in Prevalence Studies Tool (RBPS) [32] and the Appraisal Tool for Cross-Sectional Studies (AXIS) [33] were used to evaluate the reliability, validity and bias of each of the eligible studies.

### Data analysis

Prevalence data from the eligible studies were analyzed using Comprehensive Meta-Analysis Software [34]. For each mental disorder, raw data from the above-mentioned spreadsheet were used to calculate the point prevalence (number of events/sample size) of each region, to enable weighting of results. A random effects model was used to determine the random effects pooled prevalence rate of mental disorders in Europe, from studies which reported the prevalence of “any mental disorder”. A random effects model was also used to create forest plots and establish the Random Effect Pooled Prevalence Rate (REPPR) for each individual mental disorder. This model was chosen because studies included different populations with a varied effect size.

Population studies measure the number of people who are diagnosed with a disorder from the total number of individuals assessed in a sample. On the other hand, register-based studies determine the number of individuals who are registered with a disorder in the target population. Since the entire target population is not assessed in the latter, individuals who do not actively seek help from mental health professionals may remain undiagnosed. Moreover, individuals who refuse to report their diagnosis may not be included in register-based studies unless this is made mandatory. The prevalence rates obtained by the two types of methodologies are therefore very different and their populations are not homogenous. In view of this, the two types of studies were analyzed separately to avoid Simpson’s Paradox [35], which may arise when there is a significant discrepancy of factors at many levels of the variable of interest.

The Standardized Residual Values (SRV) were examined from cross-national prevalence comparisons and a cut-off of ± 3 at 95% confidence interval was used to identify outliers [36]. A One Study Removal Analysis was conducted to identify whether studies with prevalence rates that are very different to the reset are influential [36].

Prevalence rates were compared across countries, gender and level of education when this information was available by five or more studies for a specific disorder. This was not done for disorders with less studies since the analysis would be underpowered to obtain meaningful results [37]. An analysis by level of education was conducted to compare the prevalence rates of young children who attend primary school to older children who attend secondary school. It was not possible to compare prevalence according to specific age groups since the eligible studies obtained results for a varied range of age groups making them incomparable.

A meta-regression analysis to determine the contribution of specific cofactors (such as number of study phases’ response rate in screening and diagnostic phase, type of informants, etc.) to heterogeneity was not conducted because fewer than ten studies were identified for each mental disorder and therefore results would be insignificant [38].

## Results

**Fig. 1:**
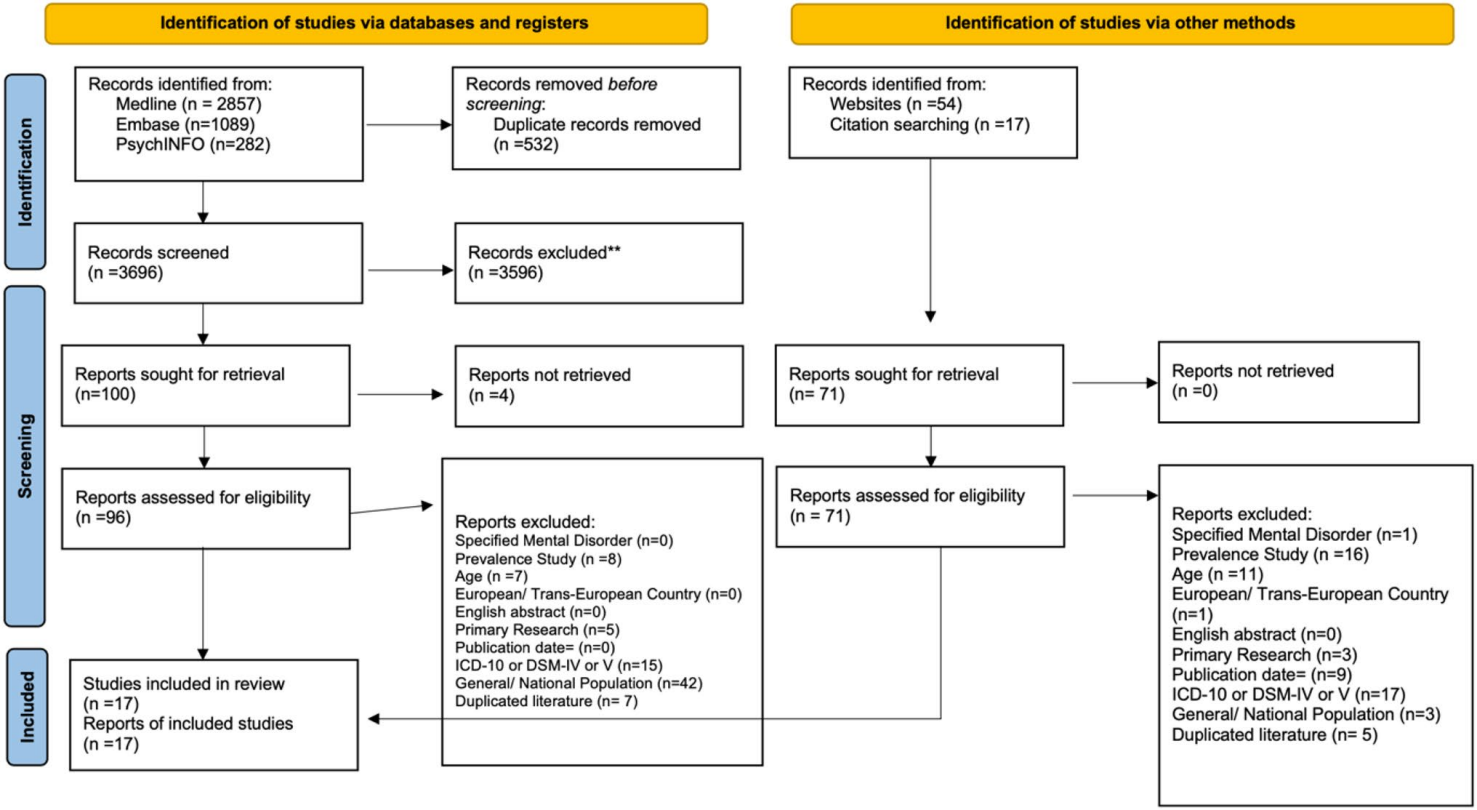
Prisma flow chart [42] illustrating the selection process

This SR identified 4228 potentially relevant articles from three databases and 59 from the gray literature. An additional 17 studies were identified from reference lists. Figure 1 shows detailed results of the number of studies included and excluded at each phase of the selection process. Seventeen studies met the inclusion criteria (Table 1), encompassing a total of 50,605 participants from fourteen European countries when considering the population studies alone.

**Table 1:** Eligible studies and their characteristics

Author year	Region [Nationwide]	Sample number (% male, % females)	Age range (years)	Level of education			Diagnostic classification	ASD	ADHD	ODD	CD	Anxiety disorder	Depressive DISORDER	Eating disorder	SUD	
				Primary	Secondary	Both										Risk of bias (ROB) scores** (AXIS, RBPS)
Boilson AM et al. (2016), [56]	Ireland (Galway, Waterford, Cork) [No]	7951 (54%, 46%)	6–11	Yes	No	No	DSM-IV TR	Yes	No	No	No	No	No	No	No	2, 1
Budisteanu M et al. (2017), [41]	Romania [Yes]	NA	7–9	Yes	No	No	DSM-IV TR	Yes	No	No	No	No	No	No	No	7, 3
Canals-Sans J et al. (2018), [50]	Spain (Reus) [No]	1510 (48%, 52%)	9–11*	Yes	No	No	DSM-V	No	No	No	No	No	Yes	No	No	3, 0
Delobel-Ayoub M et al. (2020), [55]	Denmark, Finland, Iceland, South West France, South East France [Yes]	NA	7–9	Yes	No	No	ICD-10	Yes	No	No	No	No	No	No	No	3, 3
Donfrancesco R et al. (2015), [51]	Italy (Tuscany, Latium) [No]	1887 (49%, 51%)	7–13*	Yes	Yes	Yes	DSM-IV	No	Yes	No	No	No	No	No	No	1, 1
Elberling H et al. (2016), [43]	Denmark (Copenhagen) [No]	1585 (52%, 48%)	5–7	Yes	No	No	ICD-10	Yes	Yes	Yes	Yes	Yes	Yes	No	No	0, 1
Ercan ES et al. (2016), [44]	Turkey (Izmir) [no]	419 (54%, 46%)	6–14	Yes	No	Yes	DSM-IV	No	Yes	Yes	Yes	Yes	Yes	No	No	1, 0
Lesinskiene S et al. (2018), [47]	Lithuania (Nationwide) [Yes]	526 (54%, 41%)	7–17	Yes	Yes	Yes	ICD-10	Yes	Yes	No	Yes	Yes	Yes	No	No	1, 0
López-Villalobos JA et al. (2015), [52]	Spain (Castile and Leone) [No]	1049 (52%, 48%)	6–16	Yes	Yes	Yes	DSM-IV	No	No	Yes	No	No	No	No	No	1, 2
Magklara, K. et al. (2015), [49]	Greece [Yes]	5614 (45%, 55%)	16–18	No	Yes	No	ICD-10	No	No	No	No	No	Yes	No	No	2, 1
Morales-Hidalgo P et al. (2018), [57]	Spain (Tarragona) [No]	1449 (50%, 50%)	10–12	Yes	No	No	DSM-V	Yes	No	No	No	No	No	No	No	3, 1
Narzisi A et al. (2018), [53]	Italy (Pisa) [No]	10,138 (52%, 48%)	7–9	Yes	No	No	DSM-V	Yes	No	No	No	No	No	No	No	1, 2
NHS (2018), [46]	England [Yes]	6219 (50%, 50%)	5–16	Yes	Yes	Yes	ICD-10	Yes	Yes	Yes	Yes	Yes	Yes	Yes	No	2, 1
Politis S et al. (2017), [127]	Greece [Yes]	5614 (45%, 55%)	16–18	No	Yes	Yes	ICD-10	No	No	No	No	Yes	No	No	No	2, 1
Rojo-Moreno L et al. (2015), [58]	Spain (Valencia) [No]	962 (52%, 48%)	12–16	No	Yes	No	DSM-IV	No	No	No	No	No	No	Yes	No	3, 2
Skonieczna-Żydecka K et al. (2017), [54]	Poland [No]	2514 (81%, 19%)	8–16	Yes	Yes	Yes	ICD-10	Yes	No	No	No	No	No	No	No	2, 2
Wagner G et al. (2017), [45]	Austria [Yes]	3477 (45%, 55%)	10–18	No	Yes	No	DSM-V	No	Yes	Yes	Yes	Yes	Yes	Yes	No	1, 1

*Ages were estimated from additional sources showing the age groups of children in the mentioned school grades [39, 40]

**Scores for ROB reflect the number of elements in each tool that indicate potential for bias. AXIS has a total of 20 items whereas RBPS has 10 items

### Reliability and quality assurance

The evaluations made from RBPS [32] and AXIS [33] suggest a low-level bias among the eligible studies (Supplement 2). The AXIS indicated that all studies had an overall low potential for bias, apart from one [41] which obtained a score for moderate potential for bias. A low bias score was obtained by all studies on the RBPS.

### Mental disorders in Europe

**Fig. 2:**
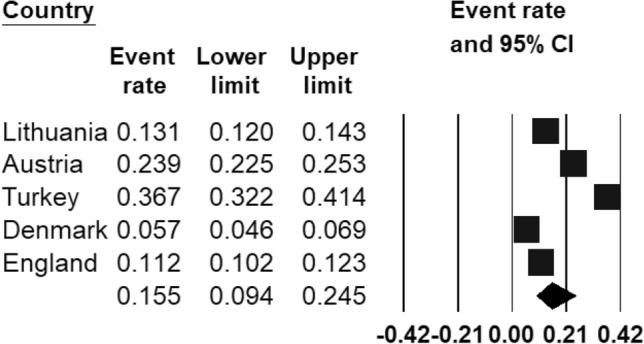
Forest plot displaying the prevalence rates of mental disorders among European regions [45–47]. (*Source*: Lithuania [47], Austria [45], Turkey [44], Denmark [43], England [46])

The pooled prevalence rate of any mental disorder (Fig. 2) ranged from 5.7% (95% CI 4.6–6.9%) in Copenhagen (Denmark) [43] to 36.7% (95% CI 32.2–41.4%) in Izmir (Turkey) [44]. Based on the eligible studies which calculated the prevalence of a range of mental disorders ([45–47]), the REPPR of any mental disorder among 5–18-year-olds in Europe was 15.5% (95% CI 9.4–24.5%, *I*^2^ = 99.8%).

### Anxiety disorders

**Fig. 3:**
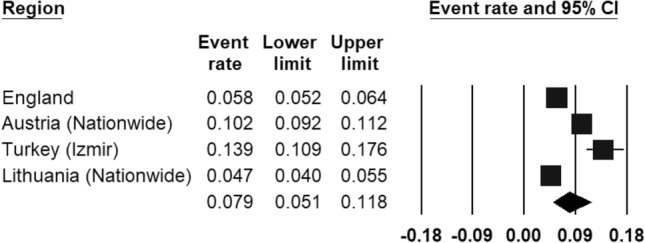
Forest plot displaying the prevalence rates of ‘any anxiety disorder’. (*Source*: England [46], Austria [45], Turkey [44]), Lithuania [47])

The prevalence of ‘any anxiety disorder’ was calculated by four population studies (Fig. 3) and ranged from 4.7% (in Lithuania [47]) to 13.9% (in Turkey [44]). The REPPR of any anxiety disorder was 7.9% (95% CI 5.1–11.8%, *I*^2^ = 98.0%). The REPPR for Agoraphobia, General Anxiety Disorder, Obsessive Compulsive Disorder and Panic Disorder ranged between 0.4 and 0.8%. Social phobia and Specific Phobia obtained a REPPR of 1.1 and 1.6% respectively. The highest prevalence rate was obtained for Social Anxiety Disorder (3.7% (95% CI 3.2–4.4%). However, this was only calculated by one study conducted in Austria [45].

### Depressive disorder

**Fig. 4:**
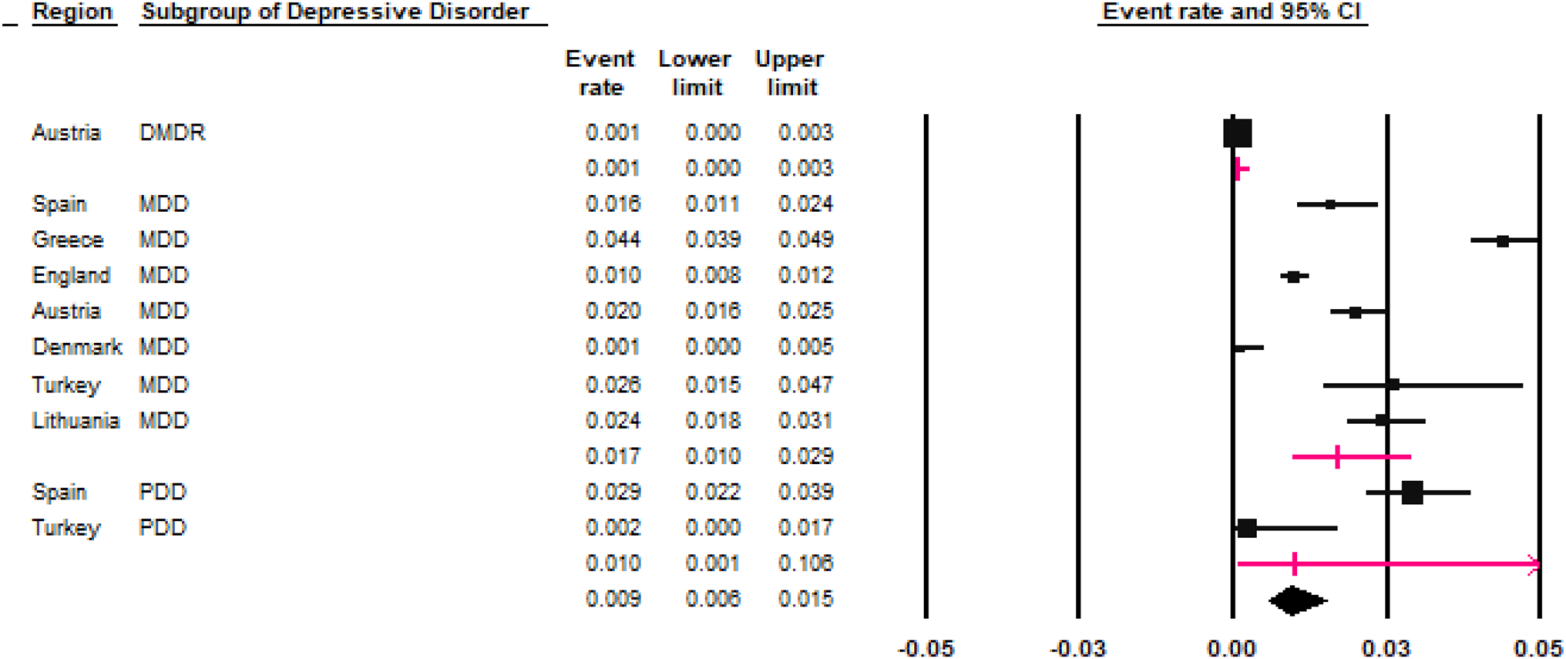
Forest plot displaying the prevalence rates of depressive disorders [45–45, 47, 49, 50]. The lines in magenta show the random effects pooled prevalence rate for each subgroup of depressive disorder. (*DMDR* disruptive mood dysregulation disorder, *PDD* persistent depressive disorder, *MDD* major depressive disorder). (*Source*: Spain [50], Greece [49], England [46], Austria [45], Denmark [43], Turkey [44]), Lithuania [47])

Two population studies estimated the prevalence of “any Depressive Disorder”, one conducted in England (1.4% (95% CI 1.2–1.7%) [48]) and one in Austria (2.8% (95% CI 1.8–3.1%) [45]). The REPPR was 2.0% (95% CI 1.0–4.0%, *I*^2^ = 97.4%) (Fig. 4). The prevalence of Major Depressive Disorder (MDD) was calculated by seven studies, obtaining an REPPR of 1.7% (95% CI 1.0–2.9%, *I*^2^ = 97.7%). The REPPR of MDD was found to be 4.2 times higher among Secondary school children (SC) (2.5% (95% CI 1.6–4.1%), *I*^2^ = 97.0%)) when compared to Primary SC (0.6% (95% CI 0.2–2.2%), *I*^2^ = 92.5%). Moreover, the REPPR among Primary SC was marginally greater for males (0.7% (95% CI 0.1–0.7%), *I*^2^ = 92.3%), when compared to females (0.4% (95% CI 0.00–4.2%), *I*^2^ = 89.8%). In Secondary SC, the opposite was found to be true, with females obtaining a REPR that was 2.15 times greater than the prevalence obtained for males.

### Attention-deficit hyperactivity disorder

**Fig. 5:**
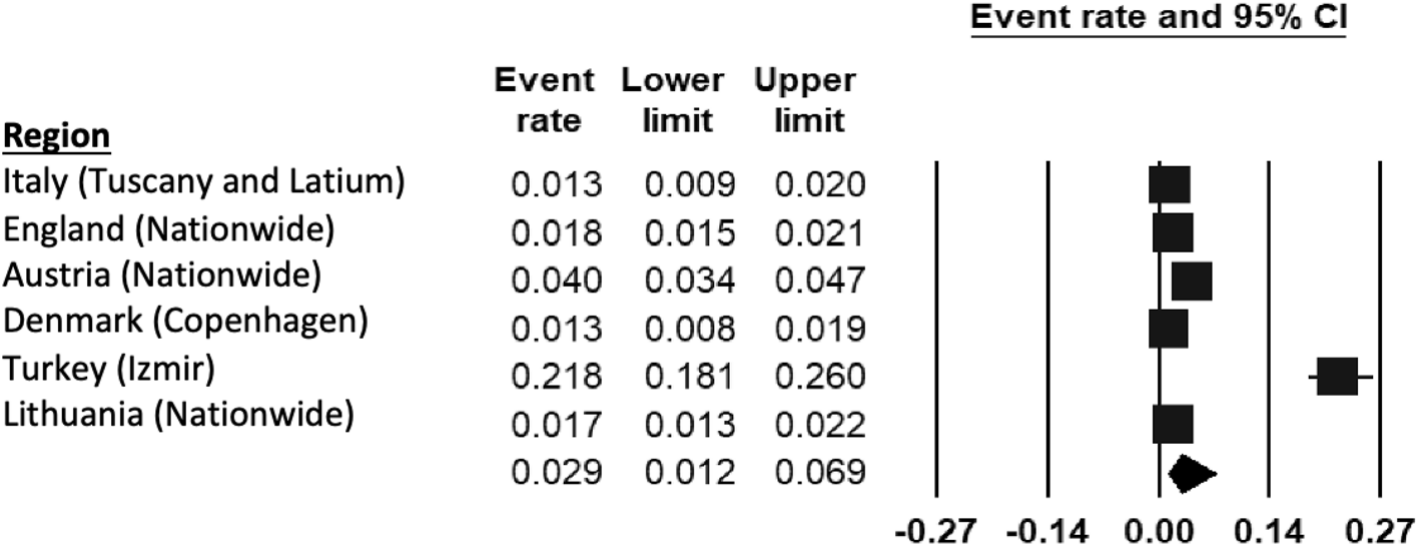
Forest plot displaying the prevalence rates of ADHD [45–47, 51]. (*Source*: Italy [51], England [46], Austria [45], Denmark [43], Turkey [44]), Lithuania [47])

Six population studies included ADHD (Fig. 5). Prevalence rates ranged from 1.3% in Tuscany, Latium (Italy) [51] and Copenhagen (Denmark) [43] to 21.8% in Izmir (Turkey) [44]. The REPPR for ADHD was calculated at 2.9% (95% CI 1.2–6.9%, *I*^2^ = 94.3%)). Moreover, the REPPR for Primary SC (3.9% (95% CI 0.9–15.7%), *I*^2^ = 99.0%) was 1.8 times higher than that of Secondary SC (2.2% (95% CI 1.2–3.8%, *I*^2^ = 95.0%). This comparison is however not statistically significant since the confidence intervals of Primary and Secondary SC overlap. Furthermore, the REPPR for males (2.3% (95% CI 1.2–3.8%, *I*^2^ = 26.9%)) was 3.3 times that of females (0.7% (95% CI 0.5–1.1%), *I*^2^ = 62.8%).

### ODD

**Fig. 6:**
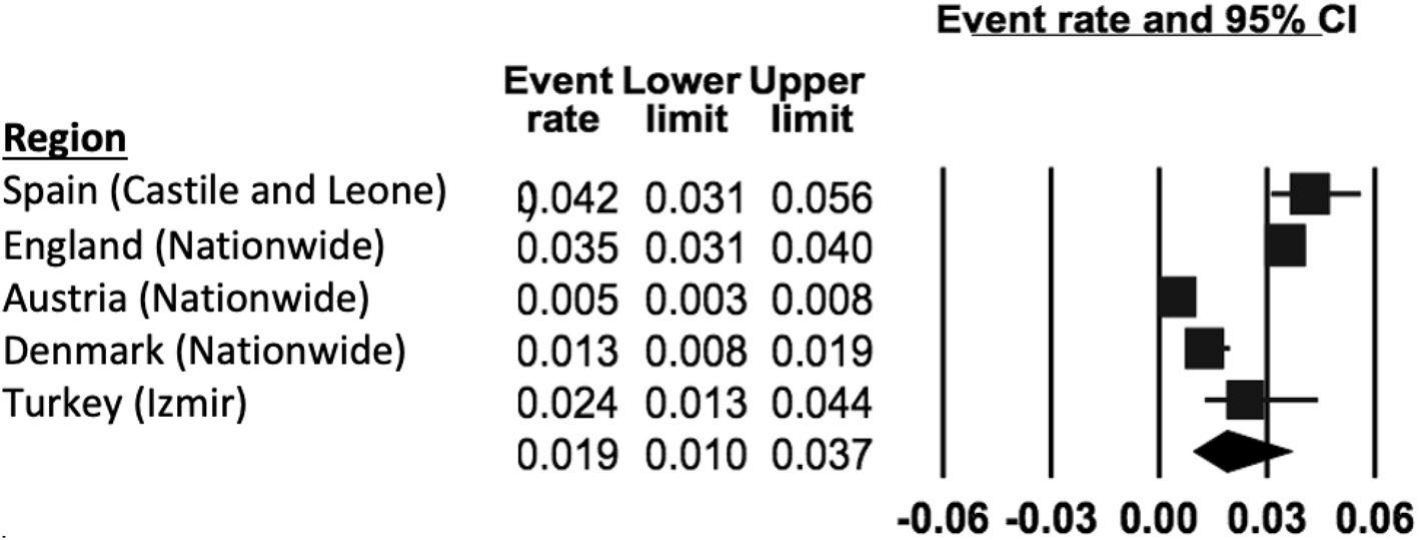
Forest plot displaying the prevalence rates of Oppositional Defiant Disorder [45–46, 52]. (*Source*: Spain [52], England [46], Austria [45], Denmark [43], Turkey [44])

The REPPR of ODD was 1.9% (95% CI 1.0–3.7%, *I*^2^ = 98.0.4%), with individual prevalence rates ranging from 0.5% in Austria [45]) to 4.2% in Spain [52]) (Fig. 6). Primary SC obtained a pooled prevalence of 2.3% (95% CI 1.1–4.6%), *I*^2^ = 90.2%), which was 1.8 times higher when compared to Secondary SC (1.3% (95% CI 0.2–8.3%) *I*^2^ = 98.3%). This comparison is not statistically significant since the confidence intervals of Primary and Secondary SC overlap. Only two studies (conducted in Castile and Leone in Spain [52] and in England [46]) provided separate prevalence for both genders, showing the REPPR for males (4.8% (95% CI 3.6–6.2%), *I*^2^ = 96.2%) being 1.8 times that of females (2.7% (95% CI 2.3–3.3%), *I*^2^ = 98.3%).

### Conduct disorder

**Fig. 7:**
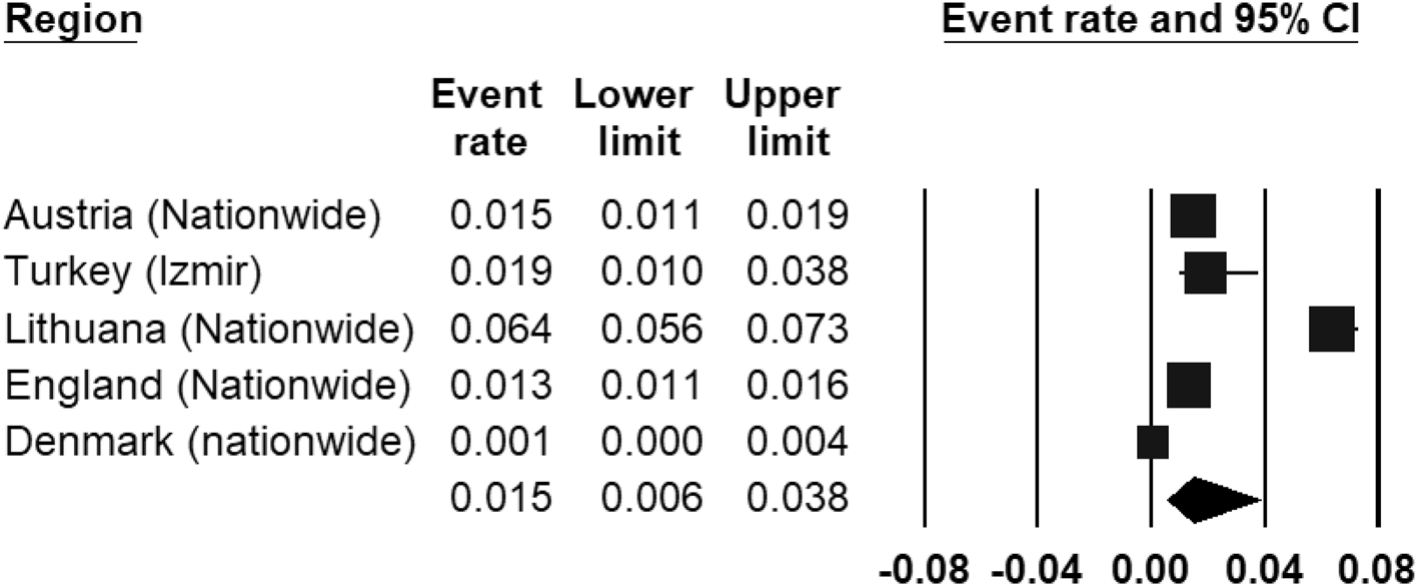
Forest plot displaying the prevalence rates of conduct disorder [45–47]. (*Source*: Austria [45], Turkey [44], Lithuania [47], England [46], Denmark [43])

The REPPR of CD was 1.5% (95% CI 0.6–3.8%, *I*^2^ = 98.8%)), with individual prevalence rates ranging from 0.1% (Denmark [43]) to 6.4% (Lithuania [47]) (Fig. 7). The REPPR of CD among Primary SC (1.2% (95% CI 0.2–5.4%), *I*^2^ = 97.5%) lower than that obtained for Secondary SC (1.7% (95% CI 0.2–5.4%), *I*^2^ = 97.3%). This comparison is not statistically significant since the confidence intervals of Primary and Secondary SC overlap. Only one study [48] compared the prevalence for both genders, showing that the prevalence for males was 1.3 times the prevalence of females.

### Autism spectrum disorder

**Fig. 8:**
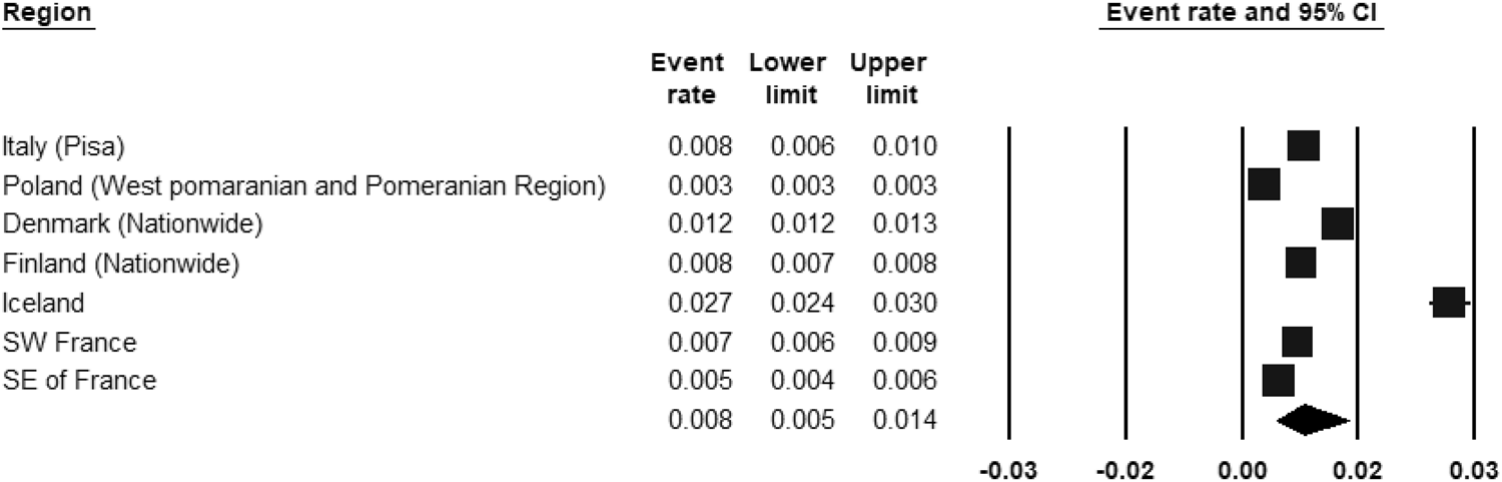
Forest plot displaying the prevalence rates of ASD from register-based studies. (*Source*: Italy [53], Poland [54], Denmark [55], Finland [55], Iceland [55], SW France [55], SE France [55])

**Fig. 9:**
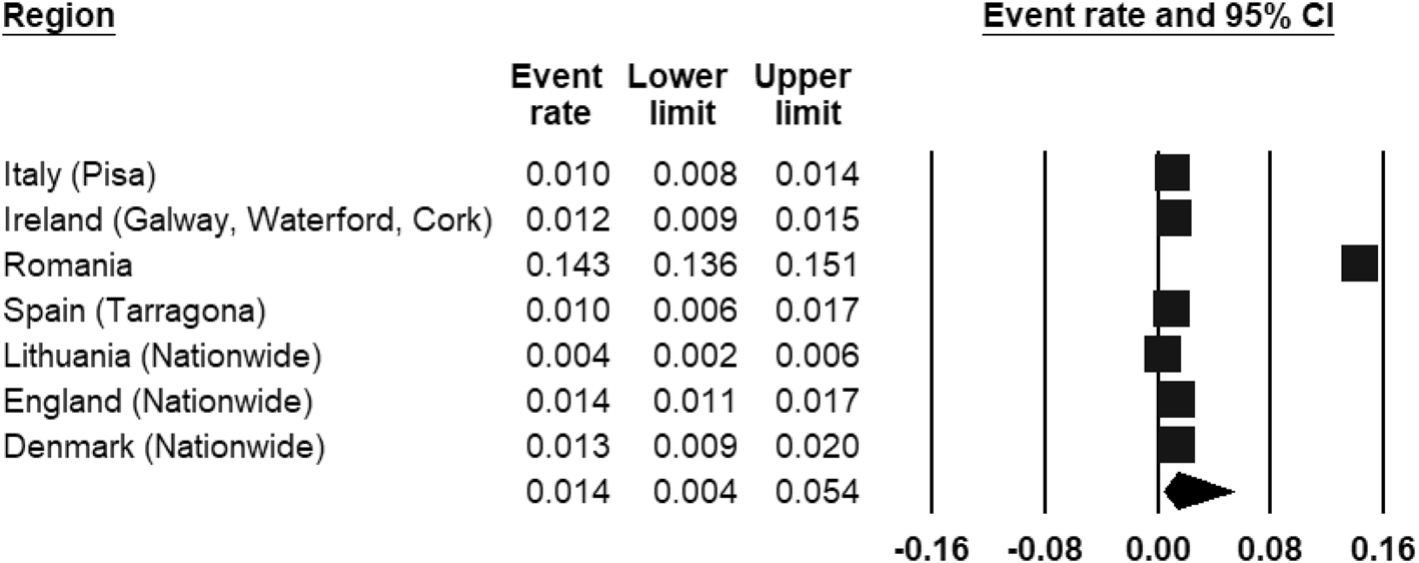
Forest plot displaying the prevalence rates of ASD from population studies. (*Source*: Italy [53], Ireland [56], Romania [41], Spain [57], Lithuania [47], England [46], Denmark [43])

ASD was included by three register-based studies (Fig. 8) and seven population-based studies (Fig. 9). The REPPR of ASD was 0.8% (95% CI 0.5–1.4%, *I*^2^ = 99.7%)) when considering register-based studies and 1.4% (95% CI 0.4–5.4%, *I*^2^ = 99.7%)) when considering population-based studies. The REPPR was 1.6% (95% CI 0.4–6.1%) (*I*^2^ = 95.5%) for Primary School Children (SC), whereas that among Secondary SC was 0.4% (95% CI 0.0–4.5%) (*I*^2^ = 92.2%), obtaining a ratio of 4:1 between the two groups. This comparison is not statistically significant since the confidence intervals of Primary and Secondary SC overlap. The REPPR of ASD for males was 2.1% (95% CI 1.7–2.5%), I^2^ = 0%), which is 3.5 times the REPPR obtained for females (0.6% (95% CI 0.4–0.9%), *I*^2^ = 0%).

### Eating disorders

**Fig. 10:**
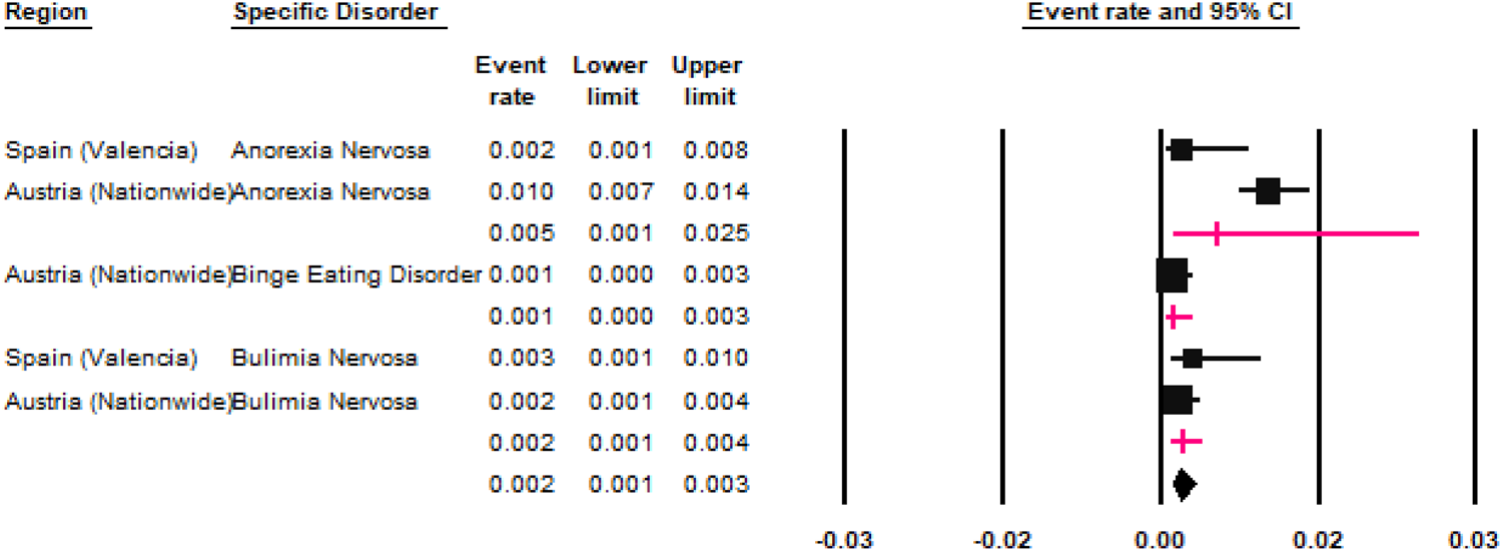
Forest plot displaying the prevalence rates of eating disorders. The lines in magenta show the random effects pooled prevalence rate for each subgroup of eating disorder. (*Source*: Spain [58], Austria [45])

The prevalence of ‘any ED’ was calculated by two studies (Fig. 10) conducted Spain [58] and England [46]; the REPPR was 1.1% (95% CI: 1.2–2.0%, *I*^2^ = 98.7%)). The studies conducted in Spain [58] and Austria [45] calculated the prevalence of AN and BN, obtaining an REPPR of 0.5% (95% CI 0.1–2.5%, *I*^2^ = 79.9%)) and 0.2% (95% CI 0.1–0.4%, *I*^2^ = 0%)) respectively. The study conducted in Austria calculated a prevalence of 0.1% (95% CI 0.00–0.7%) [45] for BED.

### Substance use disorders

None of the eligible studies calculated the prevalence of SUDs.

## Discussion

This systematic review calculated the REPPRs of mental disorders among young people in Europe, based on primary studies published between 2015 and 2020. Prevalence rates obtained by this systematic review were slightly higher than those obtained by studies published a few years ago. For instance, a study conducted across seven European countries found that 12.8% of young people have a mental disorder [59]. Similarly, another meta-analysis published in 2015 calculated that 13.4% of young people in the world may suffer from at least one mental disorder [14].

### Heterogeneity

High *I*^2^ values were obtained for most REPPRs, indicating a high degree of heterogeneity. The limited number of studies identified for each disorder precluded a meta-regression analysis that would evaluate the impact of various factors of heterogeneity. While the REPPRs need to be interpreted with caution, the narrative discussion below shall synthesize descriptive findings, describe potential factors that may influence prevalence rate discrepancies and compare results to other data in the literature.

### Anxiety disorders

Four of the six eligible studies which studied anxiety disorders calculated a REPPR of 7.9% (95% CI 5.1–11.8%, *I*^2^ = 98.0%) from a sample of 14,227 participants. This is comparable to other prevalence reported in the United States of America (USA) [60] and the global prevalence reported in 2015 [14]. This places anxiety disorders as the most prevalent mental disorder in childhood. Izmir (Turkey) obtained the highest prevalence [44] of ‘any anxiety disorder’ which may be attributed to the unfavorable social factors discussed earlier.

### Depressive disorder (DD)

DDs was calculated by two of the seven eligible studies looking at the prevalence of DD, with a REPPR of 2% (95% CI 1.0–4.0%, *I*^2^: 97.4%), which is comparable to the prevalence reported in the USA [60] and the global prevalence reported in 2015 [14]. The prevalence of major DD was reported by six studies, obtaining a REPPR of 1.7%, which is also comparable to the global prevalence [14]. Differences in the individual prevalence rates of major DD may be due to the different age groups studied. In fact, other studies have shown that major DD is relatively uncommon in pre-pubertal children [61], however it tends to increase to around 4–5% during teen years [62]. The REPPR of secondary school children is in fact 4.17 times greater when compared to that of primary school children in the eligible studies. Furthermore, socioeconomic factors may act as protective [63] and risk factors [64] to major DD and therefore contribute to the discrepancy in prevalence between different regions.

### Attention-deficit hyperactivity disorder (ADHD)

The wide variation in prevalence of ADHD, ranging from 1 to 20% shown by previous meta-analyses [65, 66] may be explained by the very different diagnostic criteria (DSM-IV, DSM-V and ICD-10) used across Europe. The prevalence for ADHD reported by the eligible studies ranged between 1.3 and 1.8%.

With the exception of the studies conducted in Italy, Austria and Izmir, the remaining studies used ICD-10 diagnostic criteria [28], which acquire a prevalence that is 4 times less than that obtained by DSM-IV and V criteria. This high proportion of eligible studies using ICD-10 criteria [28] may contribute to the lower prevalence when compared to that of 4.7% reported in Europe by another meta-analysis in which DSM-IV was most frequently used [66].

Although the study in Italy used DSM-IV criteria, it did not obtain a higher prevalence rate compared to studies using ICD-10 criteria. This may suggest a lower prevalence, which was also indicated by another meta-analysis on ADHD for Italy [67]. One possible reason for the low prevalence lies in cultural factors resulting in higher symptom tolerance, which would influence parent and teacher interpretation of the child’s behavior [67]. The study conducted in Austria was the only one to use the DSM-V [68], which has been shown to raise prevalence by 3% when compared to the DSM-IV. This may explain the higher prevalence of 4% in Austria when compared to the other studies using ICD-10 criteria [28].

The study conducted in Izmir (Turkey) [44], used DSM-IV criteria. However, the prevalence of 21.8% is much higher than prevalence rates of other areas using DSM-IV diagnostic criteria [69]. Moreover, another study conducted in Izmir using a different sample and methodology also obtained a comparably high prevalence for ADHD [70]. Although the elevated prevalence may partly be explained by the large percentage of low socioeconomic households [70] and high number of immigrants [71], which are known risk factors for ADHD [74–74], methodological differences, such as information source [74], sample demographics [75] and cultural interpretation of a child’s behavior [67] may also play a role.

Based on the eligible studies which included gender-specific prevalence rates [43, 46, 51], a ratio of 3.3:1 was obtained for males and females respectively. This gender difference is similar to ratios published by earlier meta-analyses [76, 77]. Another study has shown that males generally present with symptoms of hyperactivity, whereas females are less likely to have externalizing symptoms [78] and therefore their difficulties may be less evident, resulting in missed female cases. Another discrepancy was evident between the REPPR among Primary SC and Secondary SC, obtaining a ratio of 1.8:1. Although this comparison is not statistically significant since confidence intervals of Primary and Secondary SC overlap, it mirrors the ratio obtained by another study [79]. This trend may be explained by age-related development in the prefrontal cortex [75]. Another aspect to consider is that the diagnostic criteria may be less sensitive to the presentation of ADHD in older age groups, thereby resulting in missed cases.

### Conduct disorder (CD)

Five eligible studies with a total sample size of 15,808 obtained an REPPR of 1.5% (95% CI 0.6–3.8%, *I*^2^: 98.8%). This is lower than 3.2%, the global prevalence rate established ten years ago [80]. Reasons for this may include methodological differences (such as population demographics, diagnostic criteria, heterogeneity) as well as true change in prevalence rates over time and between regions.

In a cross-national study which included seven European countries, Lithuania obtained the highest percentage of children with probable CD [59], which mirrors the findings in this meta-analysis. This feature may be understood in the context of Lithuania having higher unemployment and poverty rates when compared to the European average [81]. These poor socioeconomic factors are associated with high prevalence of CD [82]. Conversely, the low prevalence obtained by the study in Denmark may mirror good socioeconomic factors enjoyed by the cohort [43]. Although Izmir is also characterized by unfavorable socioeconomic factors, the lower prevalence may be explained by the fact that this study only included Primary SC, and the prevalence of CD is known to increase with age [83]. When considering the prevalence of Primary SC alone, Turkey obtained the second-highest prevalence after Lithuania.

### Oppositional defiance disorder (ODD)

Based on the five eligible studies (with a total sample size of 13,692 participants), the REPPR of ODD in Europe was 1.9% (95% CI 1.0–3.7%, *I*^2^: 98.4%), which was less than the global prevalence reported in 2010 [80]. Some of this variation may be due to geographical variation, in fact one study showed that the prevalence in Western Europe is 2.3 times less than that that in America [84]. However, this finding is inconsistent [85]. Although only two eligible studies reported gender-specific prevalence rates [46, 52], the male-to-female ratio of 1.8:1 was similar to results published by another meta-analysis [85]. This prevalence difference across gender may be explained by an under-diagnosis in girls; in fact, there is increasing evidence to suggest that the presentation in girls may be different to that of boys, resulting in missed cases [86].

The prevalence among the eligible studies ranged from 0.5% in Austria [45] to 4.2% in Spain [52]. The lower prevalence in Austria may partly be explained by methodological factors. First, the study in Austria used DSM-V criteria which obtains a lower prevalence for ODD than studies using DSM-IV criteria [29]. Second, it is the only study to include only Secondary SC, who are known to have a lower prevalence than younger children [80], as reflected by the prevalence ratio obtained between Primary SC and Secondary SC in this study. The higher prevalence in Spain is contrary to what would be expected when considering that a number of favorable conditions are present, such as the higher percentage of secondary and tertiary educational attainment [87] as well as yearly median income [88] when compared to the European average [88]. Methodological factors may therefore better explain the high prevalence. In fact, the study on Spain is the only study which relied on teacher-reported symptoms alone, as opposed to the other studies that also made use of self-report and parent measures.

### Autism spectrum disorder

ASD was the only condition for which both register-based and population studies were identified. The two types of studies used different methodologies and therefore the discrepancy in REPPRs was anticipated. Register-based studies carry a high risk of under-estimation, since they depend on individuals who seek help and whose diagnosis is reported.

The lowest prevalence rate was documented in the West Pomeranian and Pomeranian regions of Poland [54]. At the time this study was carried out, a lack of awareness and stigma on ASD was reported in Poland [54], which may have prevented individuals from seeking help and being assessed for the disorder. Furthermore, the lack of enforcement in reporting new cases of ASD [54] may have contributed to under-reporting such cases. Conversely, these factors may explain the higher reported prevalence rate in Iceland, a country with more awareness of neurodevelopmental disorders among the general public [89] and improved access to diagnostic services [89].

Population studies, which included a pooled sample size of 33,579 individuals, obtained an REPPR of 1.4% (95% CI 0.4–5.4%, *I*^2^: 99.7%) for ASD. This rate was comparable to 1.85% in the United States [90], and that of “around 1%” published by Autism Europe [91]. The REPPR obtained by the population studies published since 2015, is considerably higher compared to prevalence rates of around 0.2% which were reported by studies published in the 1990s [92, 93]. This rising prevalence phenomenon may be attributable to varied study methodologies [94], modifications in diagnostic criteria [95], better detection of ASD over the years [94] and development of specialist services [94]. Another factor to consider is an actual prevalence rise [96, 97] in response to an increase in environmental risk factors for ASD [24, 25, 98]. However, more research is required to confirm this.

The study in Lithuania used a case definition based on ICD-10 criteria of “Autistic Disorder” [28]. This excludes people with Asperger’s syndrome that would meet criteria for high-functioning ASD in other studies, thereby contributing to the low prevalence rate. The study in Romania followed a standardized multi-national methodology developed by the ASDEU project, therefore the methodology may not explain its discrepancy in prevalence rate. The European Union Statistics on Income and Living Conditions released in 2020 [99] showed that Romania had 37.6% of young people at risk of poverty and social deprivation, the largest percentage when compared to other European countries. These factors were connected to a raised prevalence of ASD [102–102]. Furthermore, cultural factors which may affect social cognitive processing styles [103], may influence the rate of individuals meeting diagnostic criteria for ASD.

The 3.5:1 prevalence ratio for males and females respectively, is similar to the trend reported by another meta-analysis [104] and the DSM-V [68].

### Eating disorders (ED)

Although only two eligible studies [46, 58] calculated the prevalence of ‘any ED’, the REPPR of 1.1% (95% CI 1.2–2.0%, *I*^2^ = 98.7%) is comparable to another study conducted in the USA [105]. Similarly, the REPPR obtained for AN, BN and BED are also on par with results obtained by a global meta-analysis [106] and a population study conducted in the USA [107].

### Substance use disorders (SUD)

No eligible studies on SUD were identified. Although some studies on the prevalence of young people who used alcohol [108] and drugs [109] were identified, none of them used gold standard diagnostic criteria for SUD, with the exception of the Adolescent Brain Study [110] which was not eligible for inclusion due to its publication date. The latter study revealed that none of the young adolescents met criteria for a SUD. The present study identified a gap in the literature and encourages researchers to carry out population studies to better understand the impact of SUDs on young people in Europe. Moreover, studies on SUD among young people may not use formal diagnostic criteria because the threshold for what is considered a disorder may be set too high for this age group.

### Recommendations developed from this systematic review

A number of recommendations to improve mental health among young people in Europe are drawn from this study. Firstly, future epidemiological studies need to be enhanced in a number of ways. Multi-country prevalence studies with methodologies which are replicable and utilize the same diagnostic criteria are needed to improve comparison across countries and elicit trends over time. This has been attempted by some associations such as the ASDEU [111] for ASD, nonetheless such studies are required for all mental disorders. Moreover, the discrepancy in prevalence rates between population and register-based studies indicates that a considerable number of young people with neurodevelopmental disorders remain undiagnosed. This echoes the literature which reports that more than half of children remain undiagnosed [112] and less than 20% receive treatment [113]. To improve this, routine screening for the entire population, together with obligatory reporting is recommended. Screening programs in schools enhance early detection [114] and improve the outcomes of affected individuals [115]. Although nationwide screening is costly, long-term morbidity as a result of mental disorders is likely to outweigh the costs of adequate detection and prompt treatment [116].

Second, diagnostic sensitivity for specific groups needs to be improved. Prevalence discrepancy was noted between different genders as well as age groups. Many theories have attempted to support such discrepancies, as shown by the hypotheses of the ‘female protective effect’ [117] and the ‘extreme male brain’ for ASD [118]. However there has been a growing collection of evidence that females need a larger symptom demonstration [119] to acquire a diagnosis and are being diagnosed later than males [119]. Similarly, although neurodevelopmental disorders are life-long conditions, lower prevalence rates were obtained by older age groups, raising a query on diagnostic sensitivity.

Increased awareness and reduced stigma of childhood mental disorders are also required. Considerably different prevalence rates were obtained by register-based studies from countries with contrasting levels of stigma and lack of awareness [54, 56]. This demonstrates that stigma is a barrier to prevention and treatment strategies. Results of population studies may have been less influenced by stigma because all individuals within a sample were assessed; however, stigma may still contribute to inaccurate self-reports and lead to social desirability bias [120]. Young people, parents, teachers and general practitioners may have poor mental health literacy [121, 122] and perceive mental health problems among young people as part of normal growth and development (102, 103). This highlights the need for psychoeducation for teachers, parents, and young people to improve identification and seek appropriate support for young people with mental disorders.

The above recommendations aim to improve the identification of mental disorders, but with this comes a responsibility to treat such disorders in a timely fashion. Service development needs to be informed by current epidemiological studies which reveal the present needs. Schools, families, and parents play a key role in a child’s development. Therefore, low-cost strategies to train parents and teachers to support young people regulate emotions and react in healthy ways [123] would contribute to improved mental wellbeing. Furthermore, actions from all sectors of society need to focus on reducing socioemotional inequalities, such as poverty, unemployment, and domestic violence, to effectively improve mental health among young people. Although these recommendations are costly, they serve as an investment to improve educational outcomes, employment and productivity and thus lower costs from the criminal justice system and social benefits [124].

### Strengths and limitations

This systematic review is the first to provide an overview on the prevalence of eight MDs based on data established between 2015 and 2020, in 14 European countries. Our review includes high-quality studies with low levels of bias, that use gold standard diagnostic criteria [28, 68, 69]. Another strength lies in the comparison of prevalence rates to obtain trends across nations, gender, level of education and different time periods.

A number of limitations need to be acknowledged. First, all studies were identified and selected by one researcher, which may have resulted in selection bias and relevant studies may have been overlooked. 20% of these were checked by another researcher to diminish this. Second, prevalence rates were compared across level of education instead of age because primary studies published pooled results of a varied range of age groups. While comparison across level of education allows for certain characteristics that are specific to younger and older age groups to emerge, one must note that primary SC and secondary SC have different age groups in different countries. Another limitation lies in the fact that three diagnostic classification systems were used by the identified studies. Although these are gold standard diagnostic manuals, variations in their criteria contributed to disparities between prevalence rates of the same disorder described by different criteria.

Limitations were also caused by the low number of studies identified. Further analysis of data for gender and level of education was not possible to carry given the low number of studies identified for each disorder, which would have caused underpowered and inaccurate results. Moreover, the small number of studies limited the analysis of the effect of covariates on the prevalence rates calculated. The high I^2^ across the REPPRs shows that the observed variance reflects differences in true effect size rather than sampling error. Therefore, significant heterogeneity was present across all the random effect models. This mirrors heterogeneity obtained by other prevalence meta-analyses [14, 15]. The heterogeneity may have been attributed to sample-specific factors (such as gender, age, country, culture) and methodological factors (such as type and number of informants, number of phases, screening and diagnostic instruments, diagnostic criteria, etc.). A meta-regression analysis that includes all potential covariates would have ideally investigated the impact of individual factors to heterogeneity. However, results would not have been meaningful with a low number of studies for each covariate [38].

## Conclusion

To our knowledge, this is the most up-to-date meta-analysis that calculates the pooled prevalence of mental disorders among children and adolescents in Europe. Although 15.5% of young people in Europe were estimated to suffer from a mental disorder, one must also factor in an additional rise in prevalence since COVID-19 has increased neuropsychiatric manifestations [125] with households from lower socioeconomic factors anticipated to have had a worse outcome. Furthermore, the war between Ukraine and Russia is also expected to raise the prevalence of mental disorders among affected and neighboring countries [126], with negative consequences persisting after the war [126]. With these unprecedented risk factors for mental disorders, Europe must work more than ever before to prevent, diagnose and treat mental disorders promptly. Improving diagnostic sensitivity, developing routine screening and early intervention services, raising awareness of mental disorders, and tackling socioeconomic inequalities, contribute to a long-term investment for improved functioning of society.

## Supplementary Information

Below is the link to the electronic supplementary material.

Supplementary file1 (DOCX 30 KB)
