# Supplementary material for: A systematic review and meta-analysis on the prevalence of mental disorders among children and adolescents in Europe
Source: Eur Child Adolesc Psychiatry. 2022 Dec 30;33(9):2877–94. doi: 10.1007/s00787-022-02131-2 (PMC9800241; doi:10.1007/s00787-022-02131-2)
Supplement: Supplementary file 1 — Supplementary file1 (DOCX 30 KB) [file 787_2022_2131_MOESM1_ESM.docx]

**Supplement 1**

| SPIDER tool | Search Strings |
| --- | --- |
| Sample | child*.mp. OR adolescent/ or child/ or child, abandoned/ or child, adopted/ or child, exceptional/ or "child of impaired parents"/ or child, foster/ or child, orphaned/ or child, unwanted/ OR teen*.mp. OR young*.mp. OR adolescen*.mp. AND europ*.mp. OR europe/ or france/ or united kingdom/ or greece/ or ireland/ or italy/ or mediterranean region/ or spain/ orandorra/ or austria/ or balkan peninsula/ or belgium/ or europe, eastern/ or france/ or germany/ or gibraltar/ or greece/ or ireland/ or italy/ or liechtenstein/ or luxembourg/ or mediterranean region/ or monaco/ or netherlands/ or portugal/ or san marino/ or "scandinavian and nordic countries"/ or switzerland/ or transcaucasia/ or ussr/ or vatican city/ |
| Phenomenon of Interest | anx*.mp. OR anxiety disorders/ or agoraphobia/ or anxiety, separation/ or neurotic disorders/ or obsessive-compulsive disorder/ or panic disorder/ or phobic disorders/ or general anxiety disorder/ or social anxiety/ OR depress*.mp. OR affective symptoms/ or depression/ or self-injurious behaviour/ OR adhd.mp. OR attention deficit hyperactivity disorder.mp. or Attention Deficit Disorder with Hyperactivity/ OR conduct disorder/ or oppositional defiant disorder/ OR neurodevelopment disorders.mp. OR ASD.mp. OR child development disorders, pervasive/ or autism spectrum disorder/ or asperger syndrome/ or autistic disorder/ OR Autis*.mp. OR substance-related disorders/ or alcohol-related disorders/ or stimulant-use disorder/ or amphetamine-related disorders/ or cocaine-related disorders/ or drug overdose/ or inhalant abuse/ or sedative-use disorder/ or hyponotic-use disorder/ or marijuana abuse/ or cannabis-use disorder/ or opioid-related disorders/ or hallucinogen-use disorder/ or phencyclidine abuse/ or psychoses, substance-induced/ or substance abuse, intravenous/ or substance abuse, oral/ OR substance misuse.mp. OR alcoholism/ or binge drinking/ OR addic*.mp. eating disorder*.mp. OR "feeding and eating disorders"/ or anorexia nervosa/ or binge-eating disorder/ or bulimia nervosa/ or "feeding and eating disorders of childhood"/ or food addiction/ or night eating syndrome/ OR self-injurious behavior/ or self mutilation/ or suicide/ OR exp Suicide, Attempted/ or self harm*.mp. OR mental disorder*.mp. |
| Design | interview.mp. OR survey.mp. OR register.mp OR census.mp. |
| Evaluation | morbidity/ or incidence/ or prevalence/ or prevalen*.mp. |
| Research Type | epidemiol*.mp. |

**Supplement 2**

Table 2: Appraisal Tool for Cross-Sectional Studies (AXIS)

| Question Number | Study Number | | | | | | | | | | | | | | | | |
| --- | --- | --- | --- | --- | --- | --- | --- | --- | --- | --- | --- | --- | --- | --- | --- | --- | --- |
|  | 1 | 2 | 3 | 4 | 5 | 6 | 7 | 8 | 9 | 10 | 11 | 12 | 13 | 14 | 15 | 16 | 17 |
| 1 | N | N | N | N | N | N | N | N | N | N | N | N | N | N | N | N | N |
| 2 | N | N | N | N | N | N | N | N | N | N | N | N | N | N | N | N | N |
| 3 | N | N | N | N | N | N | N | N | N | N | N | N | N | N | N | N | N |
| 4 | N | N | N | N | N | N | N | N | N | N | N | N | N | N | N | N | N |
| 5 | N | N | B | N | B | N | N | N | N | N | N | N | N | N | N | N | N |
| 6 | N | N | N | N | N | N | N | N | N | N | N | N | N | N | N | N | N |
| 7 | N | N | N | B | N | N | N | N | B | B | B | B | N | N | N | N | N |
| 8 | N | N | B | NA | B | N | N | N | N | N | N | N | N | N | N | N | N |
| 9 | N | N | N | NA | B | NA | N | N | N | N | N | N | N | N | N | N | N |
| 10 | N | N | N | B | N | N | N | N | N | N | N | N | N | N | N | N | N |
| 11 | N | N | N | N | N | N | N | N | N | N | N | N | N | N | N | N | N |
| 12 | N | N | N | N | N | N | N | N | N | N | N | N | N | N | N | N | N |
| 13 | B | B | N | B | N | B | N | NA | N | B | N | NA | B | B | N | N | N |
| 14 | N | B | N | B | N | N | B | N | B | B | B | B | B | N | N | B | B |
| 15 | N | N | N | N | N | B | N | N | N | N | N | N | N | N | N | N | N |
| 16 | N | N | N | N | N | N | N | N | N | N | N | N | N | N | N | N | N |
| 17 | N | N | N | N | N | N | N | N | N | N | N | N | N | N | N | N | N |
| 18 | N | N | N | B | N | N | N | N | N | N | N | N | N | N | N | N | N |
| 19 | N | N | N | N | N | N | N | N | N | N | N | N | N | N | N | NA | N |
| 20 | N | N | N | N | N | N | N | N | N | N | N | N | N | N | N | NA | N |
| Total | 1 | 2 | 2 | 7 | 3 | 3 | 1 | 1 | 2 | 3 | 2 | 3 | 2 | 1 | 0 | 3 | 1 |

*(N= No bias, NA= No information provided, B= Bias elicited.*

***Numbers in the last row are the total number of cells which indicate presence of bias or no information to determine whether bias is present.***

Table 3: Risk of Bias in Prevalence Studies Tool

| Question Number | Study Number | | | | | | | | | | | | | | | | |
| --- | --- | --- | --- | --- | --- | --- | --- | --- | --- | --- | --- | --- | --- | --- | --- | --- | --- |
|  | 1 | 2 | 3 | 4 | 5 | 6 | 7 | 8 | 9 | 10 | 11 | 12 | 13 | 14 | 15 | 16 | 17 |
| 1 | N | N | N | N | N | N | N | N | N | N | N | N | N | N | N | N | N |
| 2 | N | N | B | N | B | N | N | N | N | N | N | N | N | N | N | N | N |
| 3 | N | N | N | N | N | N | N | N | B | N | B | B | N | N | N | N | N |
| 4 | B | B | N | B | N | B | N | NA | N | N | N | NA | B | B | B | N | N |
| 5 | N | N | N | N | N | N | N | B | N | N | N | N | N | N | N | N | N |
| 6 | N | N | N | N | N | N | B | N | N | N | N | N | N | N | N | N | N |
| 7 | N | N | N | NA | B | N | N | N | N | N | N | N | N | N | N | N | N |
| 8 | B | N | N | B | B | N | N | N | N | N | N | N | N | N | N | N | N |
| 9 | N | N | N | N | N | N | N | N | N | N | N | N | N | N | N | N | N |
| Overall | 2 | 1 | 2 | 3 | 3 | 1 | 1 | 2 | 1 | 0 | 1 | 2 | 1 | 1 | 1 | 0 | 0 |

*(N= No bias, NA= No information provided, B= Bias elicited.*

***Numbers in the last row are the total number of cells which indicate presence of bias or no information to determine whether bias is present.***
